# Early Detection of Acute Chest Syndrome Through Electronic Recording and Analysis of Auscultatory Percussion

**DOI:** 10.1109/JTEHM.2020.3027802

**Published:** 2020-09-30

**Authors:** Bekah Allen, Robert Molokie, Thomas J. Royston

**Affiliations:** 1 Richard and Loan Hill Department of BioengineeringUniversity of Illinois at Chicago 14681 ChicagoIL60607USA; 2 Department of MedicineUniversity of Illinois at Chicago 14681 ChicagoIL60612USA; 3 Jesse Brown VAChicagoIL60612USA

**Keywords:** Acoustic, stethoscope, percussion, lung, acute chest syndrome, sickle cell disease, machine learning, diagnosis

## Abstract

Acute chest syndrome (ACS) is the leading cause of death among people with sickle cell disease. ACS is clinically defined and diagnosed by the presence of a new pulmonary infiltrate on chest imaging with accompanying fever and respiratory symptoms like hypoxia, tachypnea, and shortness of breath. However, the characteristic chest x-ray (CXR) findings necessary for a clinical diagnosis of ACS can be difficult to detect, as is determining which patient needs a CXR. This makes early detection difficult; but it is critical in order to limit ACS severity and subsequent fatalities. This research project looks to apply percussion and auscultation techniques that can provide an immediate diagnosis of acute pulmonary conditions by using an automated standard percussive input and electronic auscultation for computational analysis of the measured signal. Measurements on sickle cell patients having ACS, vaso-occlusive crisis (VOC), and regular clinic visits (healthy) were recorded and analyzed. Average intensity of sound transmission through the chest and lungs was determined in the ACS and healthy subject groups, revealing an average of 10–14 dB decrease in sound intensity in the ACS group compared to the healthy group. A random under-sampling boosted tree classification model identified with 94% accuracy the positive ACS and healthy observations. The analysis also revealed unique measurable changes in a small number of cases clinically classified as complicated VOC, which later developed into ACS. This suggests the developed approach may also have early predictive capability, identifying patients at risk for developing ACS prior to current clinical practice.

## I. Introduction

Sickle cell disease (SCD) is a genetic disorder that affects the hemoglobin in red blood cells [1]. Those with SCD have hemoglobin molecules called hemoglobin S, which causes the red blood cells to have a sickled shape [1]. SCD is one of the most common autosomal recessive disorders affecting millions all over the world, and approximately 100,000 Americans [2]. These sickled cells lyse which can cause a decrease of red blood cells (RBCs), making the person anemic, as well as obstruct narrow blood vessels causing acute pain episodes, pulmonary hypertension, acute chest syndrome and other complications [2]. Acute chest syndrome (ACS) is the leading cause of death among adults with SCD [3], [4]. ACS is clinically defined as the presence of a new pulmonary infiltrate on chest imaging with accompanying fever, cough, tachypnea, hypoxia, and at times abdominal pain [3]. Often times acute chest syndrome presents a complex pathogenesis making it difficult to accurately identify a single cause for ACS in sickle cell patients [5]. In a study of 671 episodes of ACS, over 50% of ACS diagnosis was following a hospitalization for reasons other than ACS, with a pain crisis being the most common [3]. In other cases, ACS was precipitated by fat embolism, pulmonary infarction, and infection [3]. All of these reasons present challenges for establishing early diagnosis and an optimal treatment plan.

Given the necessary clinical symptoms for suspicion of ACS, chest x-ray (CXR) is currently the gold standard for detection of a pulmonary infiltrate of one complete lung lobe for a positive diagnosis of ACS. However, Morris *et al.* [6] states for physicians to even determine who needs CXR is unclear. In fact, considering only physical examination in afebrile patients with SCD, 61% of the ACS cases were not clinically suspected as such before a radiologic diagnosis was made [6]. Therefore, it would seem that it is necessary to include CXR in the initial evaluation of patients who present with fever, chest pain, or respiratory symptoms. However, infiltrates may not appear on CXR until 2 or 3 days later [6], when ACS frequently develops after an admission for a vaso-occlusive crisis (VOC). Thus, patients admitted to the hospital for severe VOC and respiratory symptoms with long hospital stays may require repeat CXRs throughout their convalescence to check for new infiltrates and evidence of ACS [5]. And, it is clear through simultaneous high-resolution x-ray computed tomography (CT) scans and perfusion scintigraphy that CXR underestimates the degree of pulmonary involvement. These CT and perfusion lung scans have shown various lung defects and evidence of disease that were shown to be otherwise normal with radiography [5]. Furthermore, multiple repeat CXRs over a patient’s hospital stay or otherwise CT scans expose the patient to radiation which could lead to the mutations of cells increasing the risk of cancer.

ACS is a life-threatening and complicated illness associated with multiorgan failure and distress, neurologic complications, and pulmonary and peripheral thromboembolism. Often times treatment is mainly supportive, making early detection of ACS critical for limiting its severity and preventing the development of the associated complications and subsequent fatalities. Significant research and studies have been performed retrospectively to add to the understanding of the elusiveness of the presentation of ACS and to better describe the full spectrum of the possible clinical presentations of this syndrome. For the 50% of patients with ACS that are preceded by admittance to a hospital for a VOC, ACS can be examined for in these early stages of the painful crisis [5]. For patients with ACS that had not been previously admitted to the hospital for another reason, one study sites that the most common finding upon physician evaluation was a normal examination, with the second most common finding being crepitations upon auscultation of the chest [7].

Having a simple, non-invasive, and highly sensitive method to diagnose ACS in the prodromal phase of the disease is paramount for the outcomes in those with SCD. Although there are multiple etiologies of ACS, the clinical diagnosis requires the detection of a new pulmonary infiltrate on CXR and the presence of crepitations via auscultation. Similar to ACS in terms of the diagnostic requirement of pulmonary infiltrate is pneumonia. Significant research has been done to determine alternative low cost, easy access diagnostic methods to CXR, one such method being pulmonary respiratory examination. One of the most widely used techniques when it comes to respiratory examination is percussion and auscultation which can provide immediate diagnosis of acute pulmonary conditions such as chronic obstructive pulmonary disease, pneumonia, and asthma [8]. But, when compared to chest x-ray, this technique’s specificity and sensitivity is low and subject to interobserver error [9]. A significant amount of research has highlighted acoustic differences in the chest caused by diseases of the lung using electronically recorded output signals from internal (breath sound) and external (e.g. percussive) inputs. Electronically recording the output signal and automating the external percussive input signal allows for more accurate and repeatable analysis and will decrease interobserver error. One group looked to optimize this technique through the use of a proof-of-concept non-invasive device they named the “Tabla”, which sends an automated percussive chirp input to the sternum to be recorded with a digital stethoscope placed on the back. This group was able to demonstrate with 92.3% accuracy a difference in sound transmission between healthy subjects and patients with pneumonia in a small study of eight healthy subjects and five with pneumonia [9].

In the present study this same device (Tabla) is paired with an electronic stethoscope (Eko) to analyze sound transmission in healthy sickle cell patients and those with ACS to determine if there are also changes in sound transmission in the presence of and preceding or leading up to ACS. As such, this will help to establish whether there are indeed detectable spectral-temporal differences to validate this as an alternative or complementary diagnostic method for earlier detection of ACS. As described in the methods, automated percussive response data was gathered from the University of Illinois Health Hospital sickle cell clinic. These patients were tracked longitudinally to gather healthy sickle cell data and to establish individualized patient baseline measurements as well as when the patient had an ACS episode. All data were then processed to establish if there were viable, statistically significant differences.

## II. Methods

### A. Human Subject Pool

Upon approval from the UIC Institutional Review Board (Protocol 2015-0466, 08/24/15), a human subject pool was created from patients with SCD at the sickle cell clinic at the University of Illinois Hospital and Health Sciences System. These were patients that were either being seen for routine clinic visits or those who were admitted to the hospital. Inclusion for the subject pool were subjects who have sickle cell hemoglobinopathy, were 18 years of age or older, and were able to give signed consent. Two patient groups were targeted, the healthy sickle cell cohort and the ACS cohort. The healthy sickle cell group was recorded from the doctor’s clinic visits or those with hospitalization for reasons other than ACS, and the ACS group was identified from the patient list of the rounding doctor at the time. The ACS group consisted of patients who had a clear clinical diagnosis of ACS, meaning a positive CXR, the current gold standard diagnostic test, along with accompanying symptoms of fever, chest pain, and other respiratory symptoms. Patients who were suspicious of developing ACS were also recorded, with retrospective evaluation of the electronic medical record (EMR) to determine if ACS did develop, then classifying that patient in the ACS group.

### B. Instrumentation and Recording Procedure

After obtaining informed consent from the patient, the stethoscope was placed on one of the six posterior locations making sure the patient was sitting up with the back off of the bed or chair and breathing at baseline. Electronic acoustic recordings were performed with the Eko stethoscope system and iOS Eko application. These recordings were saved on the secure Eko App for reference during post analysis and cross referencing of the EMR. The Tabla device was placed on the patient’s second intercostal space of their sternum. The Eko app recording was started and then a button on the Tabla was pressed to initiate the vibratory chirp (sinusoid with linearly increasing frequency with time) input from 100 to 1000 Hz over a 14 second period.

Upon completion of the chirp signal, the Eko recording was stopped and saved. This procedure was repeated for all six locations, being posterior apex left and right lobes, posterior middle left and right lobes, and posterior basal left and right lobes.

### C. Cross-Referencing EMR

A review of the EMR was done for each subject. Information including BMI, previous pulmonary medical history, smoking history, type of pulmonary disease for those with an ACS diagnosis, and type of VOC were documented and recorded. This information along with the acoustic signal analysis helped explain some variations and identify a few notable groups that required separate analysis. Post analysis revealed an exclusion criterion for technical reasons to be patients with a BMI greater than 30. Those already included in the study with BMI greater than 30 were removed from the analyzed subject pool. Additionally, subjects with complicated VOC, meaning the presence of either a pulmonary infiltrate or consolidation, and subjects with scarring were excluded from the healthy subject group.

**FIGURE 1.**
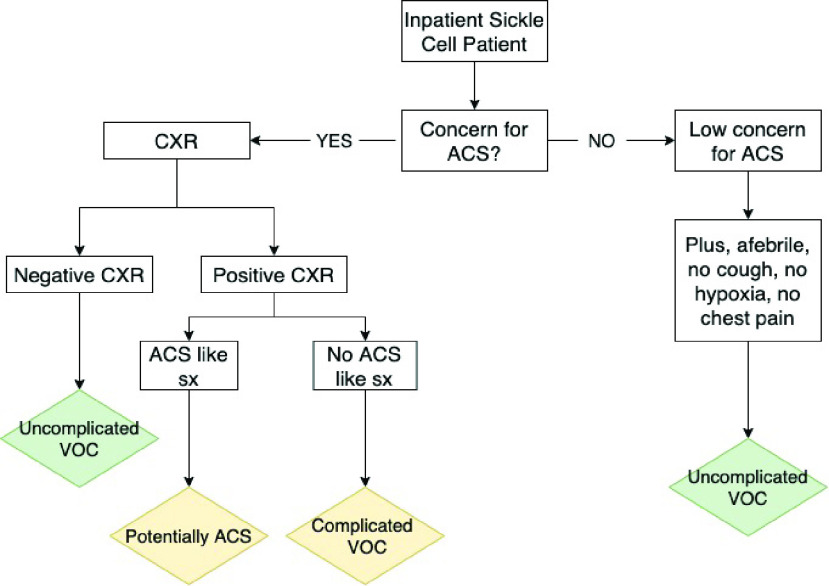
Sickle cell admit patient diagnosis pathway and EMR classification process.

**FIGURE 2.**
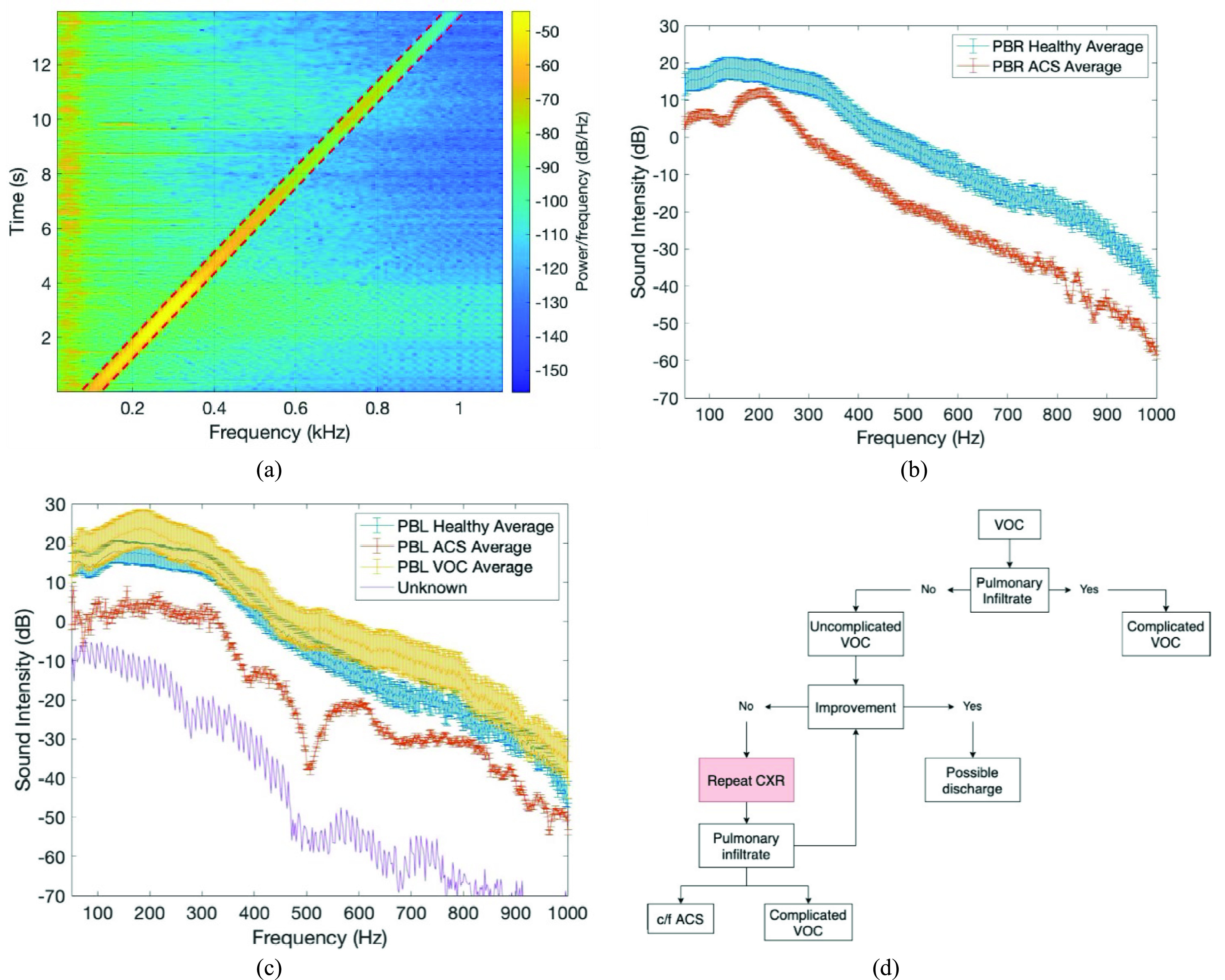
(a) Spectrogram of one of the six recording locations for the electronically recorded chirp signal transmission response from 0.1 kHz to 1 kHz over 14 seconds. (b) Average sound intensity with one standard error for the two groups: ACS (red) vs healthy (blue) at PBR. (c) Average sound intensities of subject group at PBL location, healthy (blue), ACS, (red), uncomplicated VOC (yellow), and PT 156 of unknown subject group (purple) with confounding clinical symptoms. (d) VOC in-patient potential clinical pathways. Red shaded box indicates a stage at which this system may be useful in the future.

Figure 1 explains the diagnostic pathway for sickle cell subjects that are admitted to the hospital for various reasons. Upon admission, if there is a high concern for ACS, there will be a CXR performed to determine if there is a pulmonary infiltrate to confirm the clinical definition and diagnosis of ACS, meaning both the necessary symptoms and a pulmonary infiltrate of one complete lung lobe on CXR is present. This flowchart represents a high-level overview of the clinical path for a sickle cell in-patient, but it also represents the path followed when checking the patient’s EMR for subject group classification for data analysis. The green diamonds show the diagnosis at that stage in which a subject with that path described in his/her EMR would then be added to the healthy subject group. These subjects show no signs of an infiltrate present, along with no concerning symptoms for ACS, so there would be no reason to believe that those recordings would have a decreased sound intensity. These subjects are added to the healthy subject group to train the classification model. This all holds true assuming they are discharged, and no further confounding illness develops after the initial recording. For the purpose of training a classification model, only the patients with a clear clinical diagnosis of ACS were added to the ACS subject group. However, in the future both yellow diamonds with ACS and complicated VOC have the potential to decrease the sound intensity of that recording. If a patient had followed the path of the complicated VOC diamond, then that recording was analyzed separately. An example of this is later explained in the results section in Figure 2(c).

### D. Spectral Analysis of Lung Using Automated Percussion

The data processes for the recorded 14 second chirp signal response were performed using MATLAB software (MATLAB R2016a, The MathWorks, Inc., Natick, MA). A spectrogram (estimated frequency content of a signal as it varies with time) was created for each of the six recording locations using the spectrogram function, which utilizes a short-time fast Fourier transform (FFT). The sampling rate was 4 kHz. The time record was divided into Hamming windowed 256-point blocks FFT’d to estimate the frequency content at the center time point of that block. This was done at 109 centered time points evenly spaced over the 14 seconds of the chirp signal, providing an estimate of the spectral response for every 10 Hz increment in the chirp signal as it progressed linearly with time from 20 to 1000 Hz. A moving (with time) frequency band average was taken of the spectrogram with a center frequency matching the chirp signal frequency at that point in time and the band average extending over 40 Hz (+/−20 Hz from the chirp frequency). This band is highlighted in Figure 2(a) by the red dashed lines showing the upper and lower limits of the moving average. This band was selected to capture leakage of the nonstationary chirp signal into adjacent frequencies as a result of the FFT window during which the chirp frequency would be constantly changing. The six recording locations for a single subject could then be plotted as the magnitude of the measured response (referred to as sound intensity henceforth) to the chirp input as a function of frequency.

For each of the six measurement locations, all of the healthy subjects of that lung lobe location were used to calculate the healthy group average analysis. The same was done for all of the ACS subjects per each location. In other words, all of the healthy subjects’ PBR recordings were compared to that of the ACS subjects’ PBR recordings. The average sound intensity was converted to magnitude in decibels (dB) and one standard error of the mean across the entire frequency range for each of the six locations was calculated using MATLAB’s std() function.

### E. Classification Model

A transfer learning approach was taken through supervised machine learning to begin developing an algorithm that could accurately identify and classify between healthy and ACS cases. To accomplish this, MATLAB’s Classification Learner App was employed [10]. After the data pre-processing step, the data was assigned labels based on a thorough analysis of the patient’s EMR. This analysis revealed that labeling the new training inputs required a rigorous clinical evaluation to correctly identify the two training groups, healthy and ACS, in order to develop an accurate supervised machine learning classification model.

Next, the statistical and signal processing functions available in MATLAB were used to extract 20 features such as the widely used Mel-frequency cepstral coefficients (MFCC) and other statistical measures from 145 chirp response measurements that had been processed as described in the previous sub-section. The features extracted from the isolated percussion signal were the mean, median, standard deviation, quantiles, skewness, kurtosis, and the 12 MFCC coefficients. Further feature selection was decided after performing principal component analysis and comparing predictive accuracy among different selections of features and classifiers. The selected 20 features were found to have the best performance. This architected the feature table with 20 features for 145 observations, with a labeled healthy class of 133 observations and a ACS class of 12 observations.

For this size data set, a cross-validation scheme with 5 folds was chosen for the validation scheme. The validation scheme was used to examine the predictive accuracy of the fitted models and to protect against overfitting. For a 5-fold cross validation scheme, the data gets partitioned into 5 disjoint folds, in which each fold is trained using the out-of-fold observations and model performance is evaluated with the in-fold data [11]. Finally, the average error is calculated across all of the folds, giving an estimate of the predictive accuracy of the final model trained with all of the data [11].

## III. Results & Discussion

A total of 182 subjects, 98 females and 84 males, were initially recruited from the University of Illinois Hospital and Health Sciences System. Of the 182 subjects, 11 were being treated for ACS at the time of the Tabla recording. A total of 12 subjects with a BMI greater than 30 were removed due to decreased amplitude when analyzing the mean sound transmission intensity. Three of these subjects were those with ACS, leaving a total of 8 ACS subjects with a BMI under 30. There were 58 patients that were recorded with the Eko stethoscope with a BMI under 30. Of the 58 patients included in the subject group, there were 31 females and 27 males with an average age of 37.1 years old with a standard deviation of 12.9 years. 78 subjects were not included in the final analysis due to being recorded with a Littman stethoscope which had a markedly different response as compared to the Eko stethoscope. This is not to suggest that one of these two stethoscopes is better than the other, but rather that different stethoscope models will have differences in their response dynamics that may or may not be discernable to the human ear, but certainly will create a bias in a quantitative analysis to the point that measurements from each cannot be combined, unless one can somehow weight the measured response of the one to match that of the other.

### A. Spectral Analysis of Lung Acoustics

Each subject’s recordings at each of the six lung lobe locations were analyzed individually producing the spectrogram of the chirp signal shown in Figure 2(a). Each spectral analysis was done separately for each recording site location and thus at the posterior basal right (PBR) lobe, there were 4 subjects with 5 recordings that had confirmed ACS. This average of 5 ACS recordings was compared to the 64 healthy recordings at the PBR location. Figure 2(b) shows the results of taking the signal of interest, which is the response to the chirp input and taking the moving average across the time-dependent frequency span of this signal as described previously in the methods. This analysis produced the average sound transmission intensity for the healthy and ACS subject group at the PBR location.

After a review of each subject’s recording with their EMR, it revealed interesting insights about another condition, the vaso-occlusive crisis (VOC), that occurs in sickle cell patients and often precedes the development of ACS. VOC occurs when the microcirculation is obstructed by sickled RBCs, causing ischemic injury and resulting pain. Uncomplicated VOC is diagnosed when there is an acute episode of pain without any other known cause and parenteral pain medication treatment [12]. Thus, based on the principles of sound transmission, it was not expected to confound the average sound intensity of the healthy subject pool. There were a total of 16 subjects with uncomplicated VOC specifically at the PBL location that were analyzed together to create a new group for separate analysis, the VOC group. There were 49 healthy PBL recordings and 4 ACS PBL recordings. This VOC at PBL group is shown by the yellow average in Figure 2(c) where there was not a significant difference in average sound intensity when compared to the healthy subject group.

As expected, this analysis confirmed that those subjects with a diagnosis of uncomplicated VOC for the entire duration of their hospital stay could be added to the healthy subject pool. However, review of the EMR highlighted cases of complicated VOC in which some form of pulmonary disease was reported on a CXR. But the subject did not receive a clinical diagnosis of ACS because of a lack of the other required symptoms. This distinction is important for future clinical use of this diagnostic approach, as well as to correctly classify which group a patient belongs. I.e., this system detects acoustic signals consistent with the presence of infiltrates but may require the physician to make the clinical examination to determine a diagnosis of ACS.

Retrospective analysis of the EMR and confounding results in the average sound intensity also revealed subjects that were thought to be VOC that later on developed ACS. These were patients that although did not have a diagnosis of ACS at the time, did have sound intensities similar or even more attenuated than those with ACS, before they were eventually diagnosed with ACS. This suggests the presented approach has the ability to detect ACS earlier than current clinical practice. One example is shown in Figure 2(c), where the recording was taken from an in-patient subject with a VOC diagnosis who was then discharged from the hospital. This patient was readmitted two days later and a CXR showed streaky opacities at the PBL. Figure 2(c) (unknown purple line) shows the recording taken two days prior to the positive CXR at that location which shows a decreased sound transmission intensity similar to that of the average sound intensity of the ACS group. Therefore, not having a diagnosis of ACS was not sufficient to be included in the healthy subject pool. There must not be any report of pulmonary infiltrate or disease to be classified as healthy.

Another noteworthy case is that of PT 91 who was an in-patient for a VOC with the percussive response recording taken on 7/6/18. Because of a lack of improvement by 7/21/18 a CXR was obtained but was negative. Because of the presence of a cough, SOB, and subjective fevers, on 7/26/18 a repeat CXR was obtained. This CXR introduced a new finding: a left base infiltrate vs atelectasis. Although the final discharge summary did not report a diagnosis of ACS, there was an indication of a pulmonary infiltrate that ultimately decreased the percussed sound intensity in this subject. For analysis, this subject was added to the complicated VOC category. As highlighted by this subject, at some point in a subject’s hospital stay often repeat CXRs are taken due to a lack of improvement. This is where the proposed acoustic analysis could be useful. This analysis would be useful in any stage of this subject’s hospital stay, upon admission, and/or during the first or repeat CXR. The potential for future implementation of the percussive analysis when evaluating a subject that has been admitted for VOC is outlined in Figure 2(d). While there is not a specific test to diagnose VOC, there are a number of tests that can be performed to rule out the possibility of the other complications of VOC. This percussive analysis identifies pulmonary deviations and could be used in place of or along with a CXR test. By continued validation of these methods, using this auscultatory analysis as a replacement to CXR would decrease the burden on patients, decrease radiation exposure, decrease costs, and hopefully decrease the complications associated with ACS.

For the patients that do have a pulmonary infiltrate already confirmed, but a diagnosis of VOC and not ACS, this patient has complicated VOC. However, those that do not have a pulmonary infiltrate are considered to have uncomplicated VOC. Although labeled uncomplicated VOC, the treatment and pain management is quite complex and requires multiple pharmacologic, nonpharmacologic, and preventative therapeutic interventions [13]. In the simplest cases, the patient is treated, recovers, and is discharged from the hospital. However, in some cases, the patient does not recover, and additional tests may be run to determine if new complications have developed. This would be an ideal place for the proposed percussive analysis to be used for an initial screening if there are indeed decreased sound amplitudes indicating the presence of some form of lung pathology or consolidation. Then, further CXRs, or other investigations could be ordered if one needed to visualize the exact location and type of lung infiltrate; however, it would prevent those patients who do not have any developing lung infiltrate from being exposed to additional radiation due to multiple CXRs.

**FIGURE 3.**
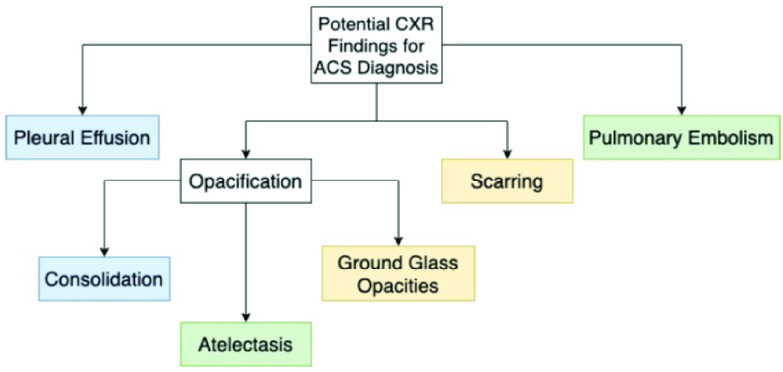
Potential findings in a positive CXR for ACS diagnosis. Blue shaded boxes suggest decreased sound transmission, green shaded boxes suggest no effect on sound transmission, and yellow shaded boxes suggest a mixed result with respect to sound transmission amplitude.

Figure 3 illustrates the various types of findings that could be present in a patient with a positive CXR and diagnosis of ACS. The findings are color coded based on how they are expected to affect sound intensity transmission. The boxes that are colored blue – pleural effusion, consolidation, and scarring – all have been shown to lower the sound transmission intensity. The lung pathology types in the green boxes have not shown to decrease sound transmission intensity due to the fact that they are not consolidative in nature; they affect the lung’s volume and vasculature and are not things that would physically change the path of sound transmission. The box with ground glass opacities (GGO) is yellow because it is a radiologic finding in computed tomography (CT) consisting of a hazy opacity that doesn’t obscure the underlying bronchial structures or vessels [14]. GGO has a broad etiology with a variety of presentations and thus in some cases it could be more consolidative and decrease the sound intensity whereas with other times when it is from a partial collapse of alveoli it would not be expected to affect the transmission of sound. These two flowcharts in Figures 1 and 3 are critical pathways to follow when correctly labeling the data inputs as healthy and ACS.

### B. Transmitted Sound Intensity Difference Between Healthy and ACS Groups

The difference in average sound intensity transmission as a function of frequency between the two subject groups was never smaller than 5 dB and on average, the difference is equal to 14.7 dB for the PBR location. For the PBL location, there is a larger deviation in difference, but again there is a similar average difference between the healthy and ACS groups of 12.8 dB.

#### 1) Classification Model

An Ensemble RUSBoosted (Random Undersampling Boosting) Tree was the chosen classifier containing 20 features and no PCA (Principal Component Analysis). This classifier performed with 94.5% positive predictive accuracy. There were other models that performed better for the healthy class but had lower accuracy for positively predicting the ACS class; this would not be acceptable.

**FIGURE 4.**
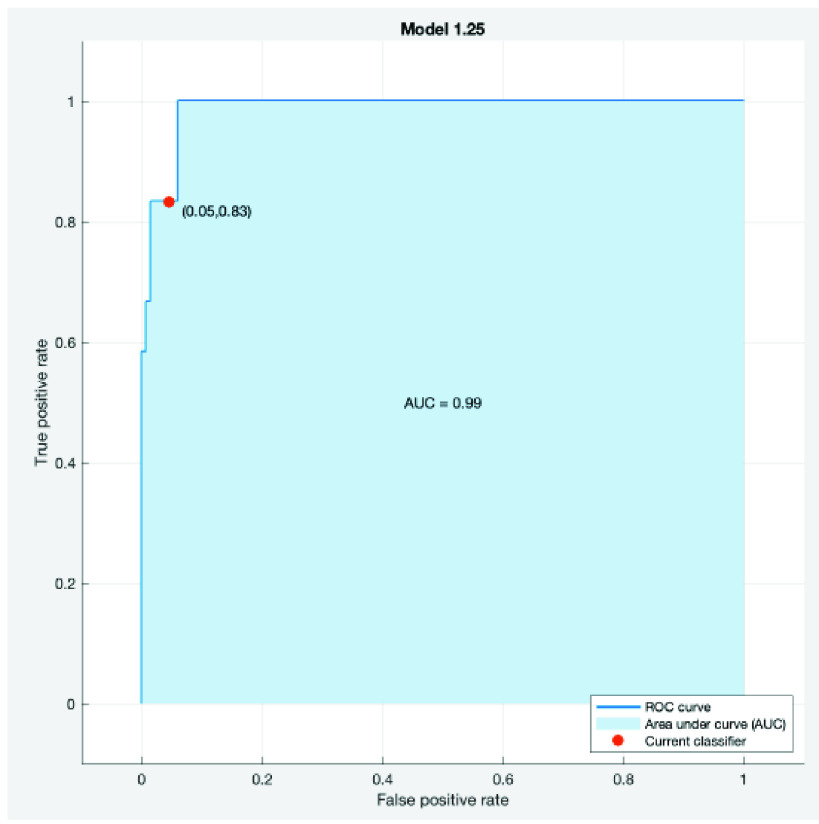
Receiver Operating Characteristic (ROC) curve for the ACS class.

This classifier type had a 0.99 AUC (Area Under the Curve) as shown in the ROC curve in Figure 4. This curve shows the true and false positive rates for the ACS class. This AUC number is quite important for the project, when considering the future clinical implementation of this entire system will require very few numbers of ACS cases to be misclassified as healthy. In clinical settings, a high false negative rate is undesirable, and can have fatal outcomes if patients are being labeled as healthy, when they do in fact have ACS. With this classifier, there is a true positive rate of 83.3%, and a 16.7% of the observations incorrectly being assigned to the positive class. More data inputs and further training the predictive accuracy of the classifier model can improve performance for future implementation.

For this classification model, the RUSBoosted tree is especially effective at classifying imbalanced data, which for this data set was the case where there were more observations in the healthy class than in the ACS class. Class imbalance is a common problem when it comes to data mining, and traditionally the two techniques employed to alleviate this problem are data sampling and boosting. Data sampling works by either oversampling which is examples added to the minority class or undersampling which is examples removed from the majority class [15]. Another technique that can improve performance regardless of class imbalance is boosting. Boosting was designed to improve the performance of any weak classifier. The most common boosting algorithm is AdaBoost which builds an ensemble of models through an iterative process [16]. In each iteration, the weights are adjusted to try to correctly classify examples in the next iteration [16]. The classification model used for this particular data is a hybrid between the two techniques in which RUSBoosting applies random undersampling. This technique randomly removes examples from the majority class and makes up for the potential of lost information through boosting. It accounts for excluded information during the construction of one model by including it in another iteration of the boosting ensemble [17].

## IV. Conclusion

Spectral analysis of the response to an electronically recorded percussive device that includes a transmission path through lung lobes indicates a difference in sound transmission intensity in the presence of certain pulmonary infiltrates. Specifically, there is a decrease of 12 – 14 dB in amplitude of the transmission spectrum in subjects with ACS as compared to healthy subjects across the entire frequency range from 100–1000 Hz. Combining these findings with a machine learning classification algorithm it was found that the approach had an overall classification accuracy of 94.5%. As more ACS data is gathered, this classification model’s true positive rate will improve for the ACS class.

An interesting secondary finding is that of the complicated VOC cases, which though not diagnosed as ACS, showed similar trends in transmission spectrum to ACS. Because VOC often precedes ACS, this finding could be important for determining early diagnostic capability of the proposed strategy. More measurements of complicated VOC are needed to confirm this capability.

What can be concluded from this study is that the lung infiltrate that presents in ACS or complicated VOC does indeed decrease sound transmission intensity compared to the sound transmission of those who are healthy. This provides clinicians with a tool to detect the presence of pulmonary infiltrates that can be used in unison with clinical examination for accurate and perhaps earlier diagnosis of acute chest syndrome.

This study demonstrates the potential of a better alternative for detection of lung infiltrates for the sickle cell patient population than the current standard of repeat CXRs for the diagnosis of ACS. Taking into consideration a decreased sound intensity suggestive of the presence of lung consolidations along with a clinical examination, this system can help aid clinicians in their diagnosis of ACS and certain cases of complicated VOC. It could also serve as a pre-validation step to determine the need for a CXR for visualization of the specific type of lung infiltrate. This also presents a better option for monitoring the progression of a pulmonary infiltrate than the high levels of radiation from multiple repeat CXRs throughout the duration of a patient’s hospital stay and over the course of one’s lifespan with sickle cell disease.

Furthering of this work includes continuing to develop the ACS subject pool and the VOC subject group. Growing the ACS data set, as well as the complicated vaso-occlusion data set, will allow additional classification models to be trained and tested. At the moment, this system does not appear to be able to differentiate between the different types of pulmonary infiltrates, and if this information is needed a CXR would still need to be performed. Further research could focus on gathering more data so that each type of pulmonary infiltrate could be analyzed separately to evaluate if there are in fact key signatures that are specific to the type of infiltrate.
